# Gut microbiota as a potential mediator of the disease-ameliorating effects of molecular hydrogen: mechanisms and therapeutic perspectives

**DOI:** 10.3389/fphar.2026.1923673

**Published:** 2026-10-23

**Authors:** Mengnan Li, Xiangrui Kong, Fanxianzi Meng, Yantong Liu, Shucun Qin, Boyan Liu, Junli Xue

**Affiliations:** Central Laboratory, The Second Affiliated Hospital of Shandong First Medical University, Tai’an, Shandong, China

**Keywords:** gut microbiota, hydrogen, inflammation, intestinal barrier, microbial metabolites, microbiota homeostasis, oxidative stress

## Abstract

Molecular hydrogen (H_2_) has emerged as a promising bioactive gas with well-documented antioxidant, anti-inflammatory, and cytoprotective properties, and its therapeutic potential for a range of human diseases has been increasingly recognized. Accumulating evidence suggests that the gut microbiota (GM) may contribute to some of the biological effects of H_2_. The GM serves as both a key site for endogenous H_2_ production and consumption, and a potential site of interaction with exogenous H_2_ supplementation, with possible implications for intestinal microecological homeostasis. This narrative review critically summarizes current evidence on the bidirectional relationship between H_2_ and the GM, focusing on microbial H_2_ production and consumption, the potential role of H_2_ in maintaining gut microbial homeostasis, and the impact of exogenous H_2_ supplementation on intestinal microecology. The potential therapeutic relevance of H_2_-associated GM modulation is further discussed in metabolic diseases, gastrointestinal disorders, and neurodegenerative diseases. Proposed mechanisms include regulation of microbial composition, reinforcement of intestinal barrier integrity, suppression of inflammation and oxidative stress, modulation of microbial metabolites, and gut-organ axis signaling. Collectively, current evidence supports that H_2_ may influence the GM ecosystem. However, most supporting data remains preclinical, and whether GM alterations causally mediate the biological or therapeutic effects of H_2_ remains to be established. Elucidating the reciprocal interaction between H_2_ and the GM may provide new mechanistic insight into H_2_-based interventions and facilitate the development of microbiota-targeted therapeutic strategies for human diseases.

## 1 Introduction

Since the initial report in 2007 in Nature Medicine highlighting the selective antioxidant effects of molecular hydrogen (H_2_), this colorless and odorless gas has rapidly emerged as a focus of biomedical research (Ohsawa et al., 2007). H_2_ is characterized by its low molecular weight, strong diffusibility, efficient biomembrane permeability, and favorable safety profile. Notably, no apparent cytotoxicity has been observed even at relatively high concentrations or under elevated pressure conditions (Abraini et al., 1994). In 2014, the U.S. Food and Drug Administration issued a “no questions” response to GRAS Notice No. 520 concerning the use of H_2_ as an ingredient in drinking water and certain beverages under specified conditions.

Over the past decade, research in hydrogen biomedicine has advanced substantially. The protective effects of H_2_ have been demonstrated in a wide range of oxidative stress-related diseases, including cardiovascular, cerebrovascular, and ischemia–reperfusion injury-related diseases (Ohsawa et al., 2008; Hayashida et al., 2008; Nie et al., 2021; Ohsawa et al., 2007; Ramanathan et al., 2023; Song et al., 2012), metabolic-hepatic-gut axis-related diseases (Kamimura et al., 2011; Liu et al., 2024; Liu et al., 2020; Takahashi et al., 2024; Zhang et al., 2015; Tao et al., 2023; Zhu Q. et al., 2025), respiratory and infection-associated inflammatory diseases (Zajac et al., 2025; Chuang et al., 2026; Yang et al., 2020; Yang et al., 2022; He et al., 2024; Xie et al., 2012), neuro-oxidative stress- and neuroinflammation-related diseases (Fu et al., 2009; Wang F. et al., 2025; Mo et al., 2026; Jiang et al., 2025; Li H. et al., 2024; Nakagawa et al., 2025), and immune-inflammatory and therapy-related tissue injuries (Wu et al., 2025; Qiu et al., 2025; Wu et al., 2026; Zhu D. D. et al., 2025; Tu et al., 2025; Yin et al., 2025; Lan et al., 2025; Zhang L. et al., 2025) (Figure 1). Notably, accumulating clinical evidence further supports the translational potential of H_2_ (Meng et al., 2025; Johnsen et al., 2023). Human studies have reported favorable effects of H_2_ in several clinically important conditions, such as metabolic syndrome (Kajiyama et al., 2008; Song et al., 2015; LeBaron et al., 2020; Liang et al., 2023; Jamialahmadi et al., 2024; Song et al., 2013), metabolic dysfunction-associated steatotic liver disease (Tao et al., 2022; Kura et al., 2022), Parkinson’s disease (Yoritaka et al., 2013), mild cognitive impairment (Nishimaki et al., 2018), hypertension (Ji et al., 2024; Liu B. et al., 2022), rheumatoid arthritis (Ishibashi et al., 2014), psoriasis (Zhu et al., 2018), COVID-19-related symptoms (Gaboreau et al., 2024; Salomez-Ihl et al., 2024), and cancer treatment-associated toxicities (Liu B. et al., 2025; Tsai et al., 2026).

**FIGURE 1:**
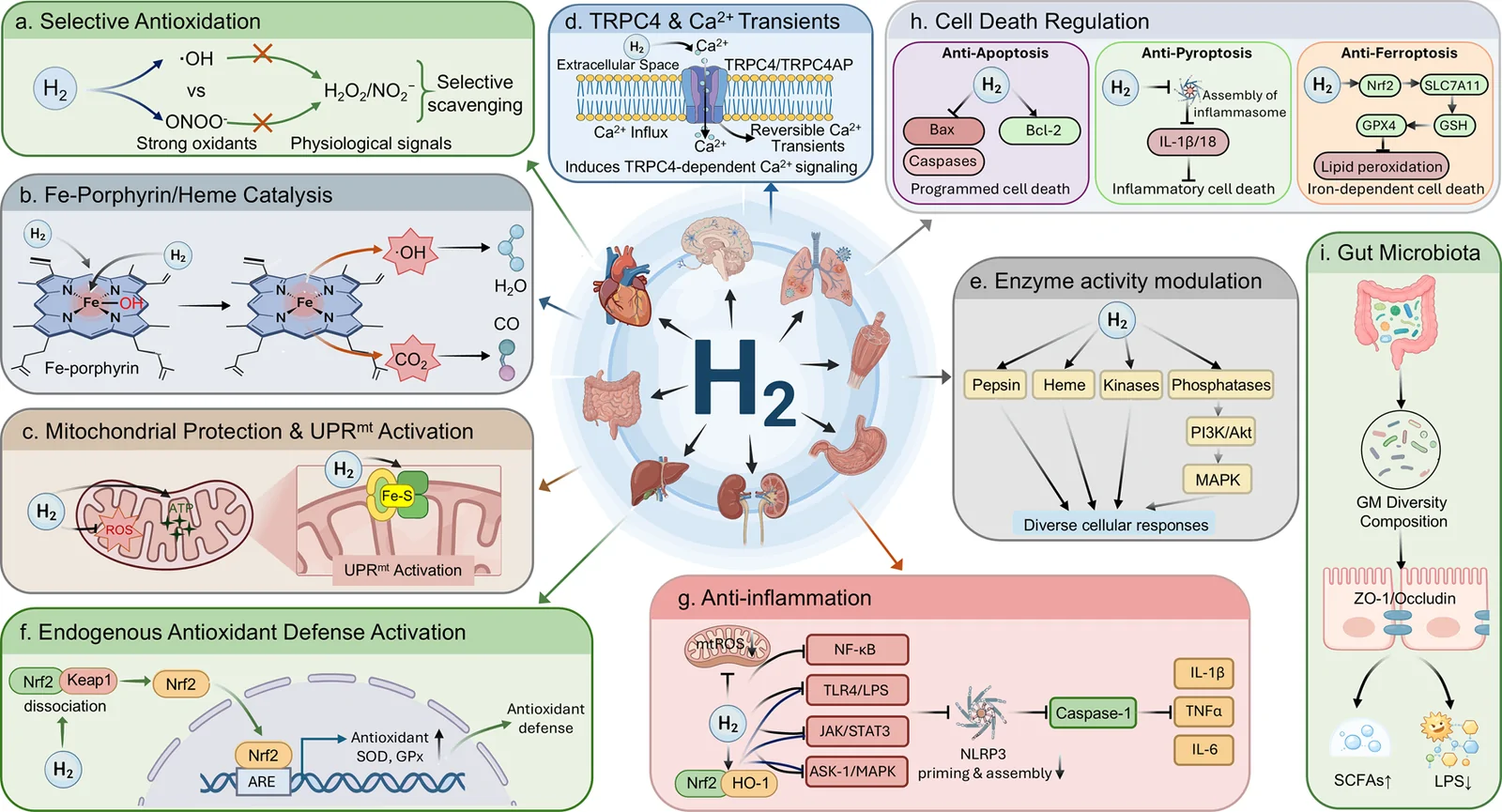
Integrated mechanistic framework for H_2_-associated multi-organ protection. Proposed H_2_-associated mechanisms include: **(a)** selective scavenging of highly reactive oxidants while preserving physiological redox signaling; **(b)** Fe-porphyrin/heme-mediated catalysis; **(c)** preservation of mitochondrial function and activation of the UPR^mt^; **(d)** induction of reversible TRPC4/TRPC4AP-associated Ca^2+^ influx; **(e)** modulation of enzyme, kinase, and phosphatase activities; **(f)** activation of Nrf2-mediated endogenous antioxidant defenses; **(g)** suppression of TLR4/NF-κB, JAK/STAT3, ASK-1/MAPK, and NLRP3-associated inflammatory signaling; **(h)** regulation of apoptosis, pyroptosis, and ferroptosis through the Bcl-2/Bax, inflammasome, and Nrf2/SLC7A11/GPX4 pathways; and **(i)** alterations in gut microbial composition and metabolites, LPS levels, and intestinal tight-junction proteins, including ZO-1 and occludin. Together, these proposed physicochemical, cellular, and microbiota-associated processes may contribute to systemic tissue protection. Abbreviations: GM, gut microbiota; GPX4, glutathione peroxidase 4; GSH, glutathione; LPS, lipopolysaccharide; NLRP3, NOD-like receptor family pyrin domain-containing 3; SCFAs, short-chain fatty acids; TRPC4, transient receptor potential canonical 4; UPR^mt^, mitochondrial unfolded protein response; Nrf2, nuclear factor erythroid 2-related factor 2.

Despite these diverse biological effects, the underlying molecular mechanisms of H_2_ remain incompletely elucidated. The early “selective antioxidant” hypothesis accounts for some of its actions. With the progression of research, this “scavenger” model alone appears insufficient to explain its broad biological activities, and its direct molecular targets remain a central issue in this field (Dixon et al., 2013) (Figure 1a). More recently, several mechanistic frameworks have been proposed, including modulation of signaling pathways, regulation of enzymatic activity, catalytic hydrogenation mediated by heme/porphyrin-containing molecules, and targeting of Rieske iron-sulfur proteins to activate mitochondrial unfolded protein response, as well as triggering reversible calcium transients *via* extracellular Ca^2+^ influx in a TRPC4-TRPC4AP-dependent manner (Negishi et al., 2025; Jin et al., 2023; Zhao et al., 2026; Ostojic, 2026) (Figure 1b-h). However, these hypotheses lack a unified consensus, and the key targets remain controversial. Growing evidence suggests that gut microbiota (GM) may represent a biological target and has been proposed to contribute to some H_2_-associated biological effects (Figure 1i).

The GM, often referred to as the second genome of the human body, comprises a highly diverse community of microorganisms and plays a pivotal role in nutrient metabolism, immune regulation, and intestinal barrier protection (Guinane and Cotter, 2013). Through metabolites such as short-chain fatty acids (SCFAs), bile acids (BAs), and neurotransmitter precursors, the GM establishes a complex bidirectional regulatory network with the host, thereby contributing to essential physiological processes including energy metabolism, immune homeostasis, and neural signaling (Krautkramer et al., 2021; O’Riordan et al., 2025). Numerous studies have linked gut microbial dysbiosis to the pathogenesis of a wide range of diseases, including metabolic disorders such as obesity and diabetes, neuropsychiatric disorders such as depression and Alzheimer’s disease, and immune-mediated diseases such as inflammatory bowel disease and rheumatoid arthritis (Guo et al., 2020; Danneskiold-Samsøe et al., 2019). Notably, the intestine serves not only as a crucial digestive organ but also as a key production factory and potential target organ for H_2_. Certain bacterial genera, such as *Bacteroides* and *Bifidobacterium*, generate H_2_ through the fermentation of dietary fiber, representing a major source of endogenous H_2_ (Tozzi and Minella, 2024). Additionally, exogenous H_2_ interventions have been associated with changes in the composition and function of the GM, together with improvements in disease-related outcomes. For example, H_2_ intervention was associated with alterations in gut microbial composition and microbial metabolites, including SCFAs, in mice (Higashimura et al., 2018). In metabolic disease models, H_2_ treatment has also been associated with increased abundance of beneficial bacteria, reduced potentially pathogenic taxa, alongside improvements in metabolic abnormalities (Qiu et al., 2020; Jin et al., 2021). In a mouse model of experimental colitis, H_2_ alleviated intestinal inflammation, with microbiota-depletion experiments supporting a contributory role of GM modulation (Ge et al., 2022). Collectively, these findings suggest that the microbiota-host axis may contribute to some of the biological effects associated with H_2_.

This narrative review synthesizes recent advances concerning the relationships among H_2_ exposure, GM alterations, and disease-related outcomes. Particular emphasis is placed on reported changes in microbial composition, functional capacity, and microbiota-host interactions. This work aims to provide a theoretical framework to support mechanistic investigations and facilitate the clinical translation of H_2_ in disease prevention and treatment.

## 2 Hydrogen metabolism and gut microbiota

### 2.1 Hydrogen production

H_2_ production in the intestinal tract is a significant by-product of intestinal microbial fermentation metabolism and mainly depends on the metabolic activities of specific microorganisms. After the ingestion of foods rich in dietary fiber, oligosaccharides, and similar components, these substrates escape digestion by host enzymes and pass into the colon, where they serve as nutrient sources for the GM (Zhang et al., 2012). Under strict anaerobic conditions, H_2_-producing microorganisms, including members of *Bacteroides*, *Bifidobacterium*, *Eubacterium*, and *Clostridium*, metabolize these carbohydrates through endogenous hydrogenase systems, ultimately generating H_2_ (Yaghoubi et al., 2024; Benoit et al., 2020) (Figure 2a). Therefore, H_2_ represents not only a major by-product of microbial carbohydrate fermentation, but also an integral component of energy flow within the intestinal microecosystem.

**FIGURE 2:**
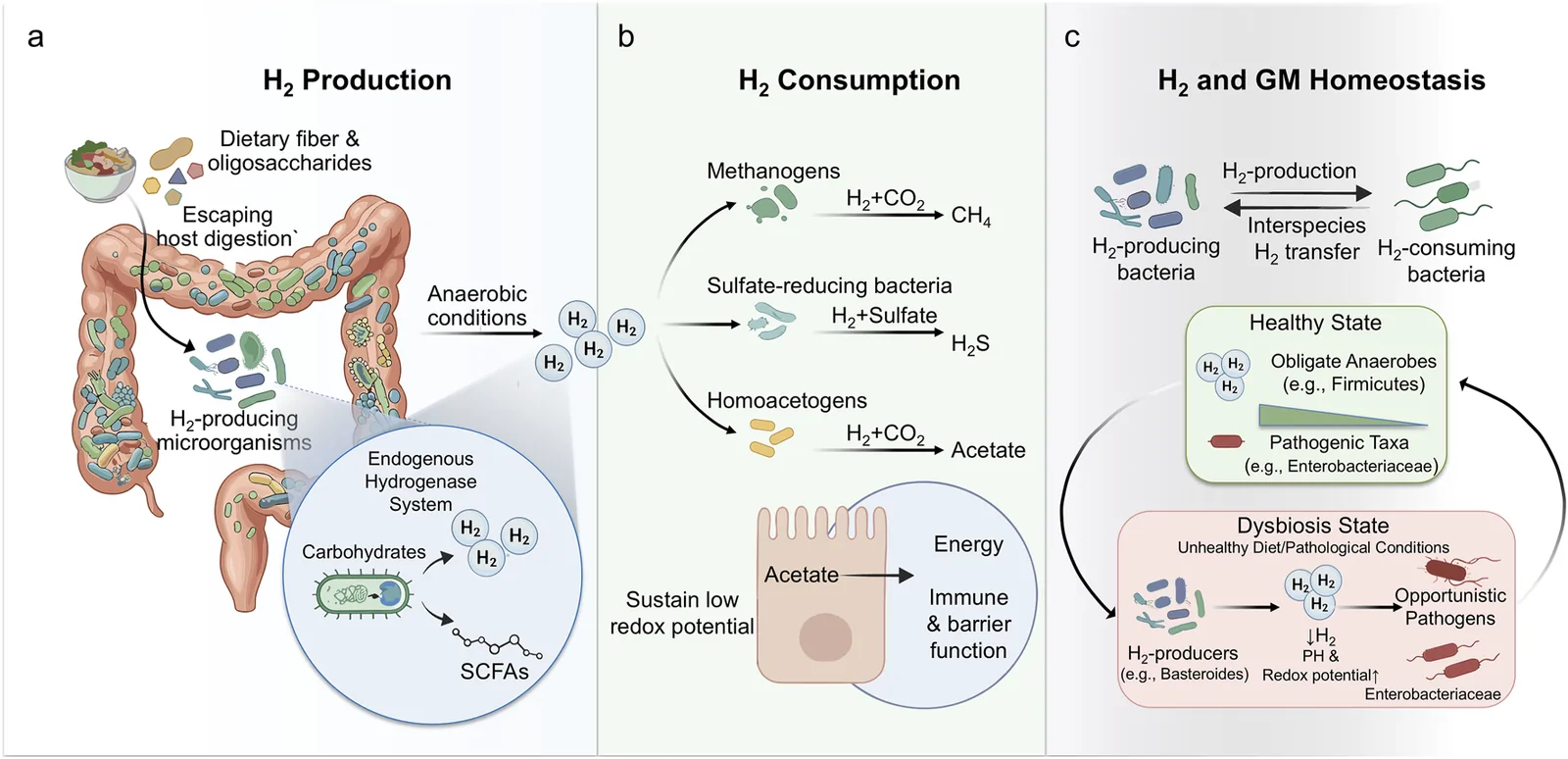
Intestinal H_2_ metabolism and gut microbial homeostasis. **(a)** Dietary fibers and oligosaccharides escaping host digestion are fermented by anaerobic microorganisms, generating endogenous H_2_ and short-chain fatty acids. **(b)** H_2_ is consumed by methanogens, sulfate-reducing bacteria, and homoacetogens to produce CH_4_, H_2_S, and acetate, respectively. These hydrogenotrophic processes may help sustain a low-redox intestinal environment, while acetate may contribute to epithelial energy metabolism, immune regulation, and barrier function. **(c)** Balanced H_2_ production, consumption, and interspecies transfer support an obligate anaerobe-dominated microbial community. Disruption of this balance under unhealthy dietary or pathological conditions may alter intestinal redox status and promote the expansion of opportunistic pathogens.

### 2.2 Hydrogen consumption

H_2_ produced in the gut is not merely a terminal product of microbial metabolism but also serves as a crucial metabolic intermediate involved in various microbial processes. Studies have shown that microorganisms capable of utilizing H_2_ as a metabolic substrate mainly include methanogens, sulfate-reducing bacteria, and homoacetogens (Birg et al., 2023). Methanogens utilize H_2_ and carbon dioxide as substrates and convert them into methane (CH_4_) through the methanogenesis pathway (Shima et al., 2020). This process not only fulfills their own energy requirements but also helps maintain a low redox potential in the gut through continuous H_2_ consumption. Sulfate-reducing bacteria use H_2_ as an electron donor to reduce sulfate to hydrogen sulfide (H_2_S) (Santos and Johnson, 2022), thereby playing a crucial role in intestinal sulfur metabolism. Homoacetogens can convert H_2_ and carbon dioxide into acetate (Zhou et al., 2024). As an important SCFA, acetate not only provides energy for intestinal epithelial cells but also participates in the regulation of intestinal immune and barrier function (Macfarlane and Macfarlane, 2003; Fukuda et al., 2011; Peng et al., 2009) (Figure 2b).

From the perspective of energy flow, the production and utilization of H_2_ constitute an essential component of the gut microbial metabolic network. H_2_-producing microorganisms convert the chemical energy stored in substrates into H_2_, whereas hydrogenotrophic microorganisms subsequently transform the energy contained in H_2_ into biologically usable forms such as ATP (Campbell et al., 2023; Schoelmerich and Müller, 2020; Cai et al., 2011). This process of energy transfer not only enhances the overall efficiency of nutrient utilization, but also contributes to the maintenance of redox homeostasis in the gut, thereby providing a favorable environment for anaerobic microorganisms and supporting the energy balance of the intestinal microecosystem (Li J. et al., 2024).

### 2.3 Clinical relevance of intestinal hydrogen metabolism and gut microbiota homeostasis

In clinical practice, H_2_/CH_4_ breath testing is commonly used as a noninvasive, indirect assessment of intestinal microbial fermentation in suspected small intestinal bacterial overgrowth (SIBO) and intestinal methanogen overgrowth (IMO) (Pimentel et al., 2020; Rezaie et al., 2017). Methanogenic archaea, sulfate-reducing bacteria, and acetogens consume H_2_ to generate CH_4_, H_2_S, and acetate, respectively. Therefore, low breath H_2_ may reflect enhanced H_2_ consumption rather than reduced fermentation (Villanueva-Millan et al., 2022). Importantly, endogenous H_2_ is continuously generated and metabolized within the intestinal lumen, whereas exogenous H_2_ produces route- and time-dependent exposure and may exert local or systemic effects. Breath-gas profiles should therefore not be considered direct indicators of therapeutic hydrogen exposure or efficacy.

H_2_-producing and H_2_-consuming microorganisms form interconnected ecological network through H_2_ metabolism (Harindintwali et al., 2023). Rapid H_2_ consumption lowers its partial pressure and supports continued fermentation, whereas H_2_-producing bacteria compete with non-H_2_-producing and non-H_2_-consuming microbes for limited carbohydrate substrate resources (Simrén and Stotzer, 2006). Additionally, H_2_ and other metabolites generated by H_2_ producers, including SCFAs, can influence the growth environment of other microbial communities by altering intestinal pH and redox potential, thereby indirectly regulating their growth and metabolic activities (Chen Q. et al., 2024). Balanced H_2_ production and consumption may therefore contribute to the metabolic stability of the GM and indirectly support intestinal barrier and mucosal immune homeostasis.

Notably, H_2_ and gut microbial homeostasis are regulated in a bidirectional manner. On the one hand, fluctuations in intestinal H_2_ levels can directly influence microbial composition and function. By lowering the intestinal redox potential, H_2_ creates favorable conditions for obligate anaerobes while limiting the expansion of aerobic and facultatively anaerobic opportunistic pathogens. For example, in a goat rumen model, increased H_2_ availability was associated with a higher abundance of Firmicutes and a concomitant reduction in potentially pathogenic taxa, including Enterobacteriaceae and *Ruminococcus* (Wang et al., 2017). Additionally, downstream metabolites of H_2_ metabolism, such as CH_4_, H_2_S, and acetate, may further reshape microbial niche distribution and community structure by altering local physicochemical properties, including pH and osmotic pressure (Jahng et al., 2012; Dardi et al., 2025; Macia et al., 2015; Xu et al., 2019). On the other hand, the composition and metabolic activity of the GM can reciprocally regulate H_2_ production and utilization. Under pathological conditions or during prolonged unhealthy dietary exposure, shifts in the abundance of H_2_-producing or H_2_-consuming microorganisms may directly alter both the amount of H_2_ generated and the metabolic pathways. For instance, female methamphetamine users have been reported to exhibit reduced levels of H_2_-producing genera such as *Bacteroides* and *Roseburia*, together with marked alterations in overall gut microbial composition, suggesting that disturbed H_2_ metabolism may profoundly impair gut microecological balance (Wang et al., 2023). Likewise, insufficient carbohydrate intake may limit substrate availability for H_2_-producing microorganisms and thereby suppress H_2_ generation (Xue et al., 2024). Disruption of H_2_ production and metabolism may therefore exacerbate microbial dysbiosis, ultimately creating a self-reinforcing cycle of dysbiosis, disordered H_2_ metabolism, and progressive impairment of gut microecological homeostasis (Figure 2c).

Taken together, microbial H_2_ production and consumption maintain a dynamic intestinal H_2_ balance that is shaped by diet, microbial community structure, intestinal transit, and disease status. Exogenous supplementation offers an alternative source of H_2_ exposure through oral hydrogen-rich water (HRW), H_2_ inhalation, rectal administration of H_2_-rich solutions, and parenteral H_2_-rich saline (HRS). As each route produces a distinct spatial and temporal exposure profile, their pharmacological properties, potential clinical indications, and translational limitations are evaluated in Section 5.

## 3 Hydrogen-induced gut microbiota alterations and potential disease-relieving effects

The GM, increasingly recognized as a functional microbial organ, is closely associated with the occurrence and progression of various diseases when homeostasis is disrupted (Huang et al., 2025). Dietary adjustments, as well as probiotic and prebiotic supplementation have been reported to modulate the GM and improve disease-related outcomes (Belizário et al., 2018). Furthermore, emerging microbiota-targeted therapies such as fecal microbiota transplantation (FMT) have demonstrated substantial clinical efficacy. For instance, in intestinal disorders, FMT has achieved cure rates of up to 98% in recurrent *Clostridioides difficile* infection (Brandt et al., 2012), highlighting the therapeutic value of targeting the gut microecology. Recent studies further indicate that H_2_ administration has likewise been associated with changes in gut microbial composition and metabolic activities in a growing number of experimental studies (Table 1). To distinguish disease-specific evidence from mechanistic interpretation, this section focuses on the disease phenotypes affected by H_2_, the accompanying GM-related changes, and the strength of evidence supporting microbiota involvement. We distinguish different levels of evidence for microbiota involvement. Parallel changes in disease phenotypes, microbial composition, or metabolites are considered associative, whereas antibiotic depletion or FMT provides stronger, intervention-supported evidence. However, these approaches do not by themselves establish microbiota-mediated causality, which would require more targeted demonstration of necessity and/or sufficiency.

**TABLE 1:** Summary of clinical and preclinical studies investigating molecular hydrogen, gut microbiota alterations, and disease-related outcomes.

Disease	Evidence type	Evidence category	H_2_ intervention	Main GM-related alterations	Interpretation of GM involvement	Disease-related outcomes	Ref.
Obesity	Preclinical—high-fat diet-fed C57BL/6J mice	Category II	HRS (oral gavage)	Partial normalization of GM composition. ↑*Bacteroides, Bifidobacteria* and *Lactobacillus.* ↓*Enterobacter cloacae*	GM remodeling, improved mucosal integrity, and reduced microbial glyoxylate-cycle activity were associated with the observed metabolic effects.	Reduced body and hepatic fat accumulation, improved lipid metabolism, mucosal pathology, and oxidative-stress indices.	Qiu et al., 2020
Metabolic disorder	Preclinical—C57BL/6N and db/db mice	Category II	Sustained-release H_2_ nanocapsules	↑*Akkermansia muciniphila*. Partial improvement in microbial homeostasis and ↓Firmicutes/Bacteroidetes ratio.	*A. muciniphila* enrichment may support intestinal barrier function and glucose metabolism.	Improved body weight, glycemia, lipid profiles, hepatic steatosis, and liver function, without reported tissue toxicity.	Jin et al., 2021
Prediabetes (IFG)	Clinical—randomized, double-blind, placebo-controlled trial; 73 participants with IFG	Category II	HRW for 8 weeks	↑*Dechloromonas* and *Flavobacterium*. ↓*Acetobacter*. Broader changes in GM composition were observed.	Associations between altered taxa and nine metabolites suggest a possible GM-metabolite-host contribution.	Reduced fasting blood glucose and visceral fat area, with improvements in oxidative-stress and inflammatory indices.	Liang et al., 2023
Atherosclerosis	Preclinical—ApoE^−/−^ mice; antibiotic depletion and FMT experiments	Category III	HRW	GM remodeling with ↑SCFA production, particularly propionate.	Antibiotic depletion and FMT findings support a microbiota-dependent contribution involving propionate and macrophage responses.	Reduced atherosclerotic lesion formation, improved plaque stability, and reduced pro-inflammatory M1 macrophage polarization.	Meng et al., 2026
MASLD	Preclinical—high-fat diet-fed Sprague-Dawley rats	Category II	H_2_ inhalation	↑Bacteroidetes and beneficial genera. ↑Bacteroidetes/Firmicutes ratio.	Improved barrier-related indices and lower circulating LPS may be linked to reduced LPS/TLR4/NF-κB signaling.	Alleviated hepatic steatosis and liver injury, with reduced inflammation.	Xue et al., 2023
MASLD	Preclinical—high-fat diet-fed C57BL/6 mice	Category II	H_2_ inhalation	↑*Akkermansia muciniphila*. ↓*Faecalibaculum* and other pro-inflammatory bacteria.	*A. muciniphila* enrichment, improved barrier integrity, and lower LPS exposure may contribute to reduced inflammation.	Lower blood glucose and lipids, improved liver function, and reduced hepatic fat accumulation.	Wang et al., 2024
ALD	Preclinical—C57BL/6J mice; FMT experiments	Category III	H_2_ inhalation	↑Lachnospiraceae, Clostridia, Lactobacillales. ↓*Prevotella*ceae, Muribaculaceae, *Alistipes*.	FMT findings support a GM-related contribution involving barrier function, LPS release, and LPS/TLR4/NF-κB signaling.	Attenuated liver injury and steatosis, oxidative stress, and inflammation.	Liu et al., 2023
Chronic ulcerative colitis	Preclinical—DSS-induced colitis in C57BL/6 mice	Category II	HRW	↓*Enterococcus faecalis*, *Clostridium perfringens*, *Bacteroides fragilis*, and *Escherichia coli*.	Suppression of potentially pathogenic taxa and changes in interferon-stimulated genes may accompany partial restoration of microbial homeostasis.	Reduced colitis severity, inflammation, and oxidative stress.	Song et al., 2022
Acute ulcerative colitis	Preclinical—experimental colitis model	Category II	HRS	↑SCFA-producing bacteria, including *Eubacterium ventriosum*, *Roseburia hominis*, and *R. faecis*, with increased SCFA production; reduced mucosa-associated *Akkermansia muciniphila* and *Ruminococcus gnavus*; restricted Enterobacteriaceae expansion	Concurrent changes in GM, SCFAs, PPAR-γ signaling, colonocyte metabolism and epithelial hypoxia are consistent with a putative H_2_–GM–SCFA–PPAR-γ pathway. Reduced mucolytic taxa may support mucus integrity, although taxa-specific mediation was not demonstrated.	Reduced colitis severity and improved mucus structure, tight-junction expression, and intestinal barrier function.	Ge et al., 2022
Ulcerative colitis—human study	Clinical—observational study—shotgun metagenomic data reanalysis	Category I	Endogenous H_2_ metabolism	↑Genes encoding hydrogenotrophic hydrogenases.	Intestinal inflammation may disrupt microbial H_2_ cross-feeding networks and favor organisms capable of utilizing H_2_ as an electron donor.	Demonstrated an association between altered microbial H_2_ metabolism and UC	Hughes et al., 2021
Ulcerative colitis—Animal study	Preclinical—mechanistic study using bacterial hydrogenase-deficient mutants	Category I	Genetic manipulation of bacterial Hyd-1 and Hyd-2, with 5% H_2_ used only in vitro	H_2_-utilizing wild-type *E. coli* outcompeted Hyd-1/Hyd-2-deficient mutants and showed enhanced expansion in the inflamed intestine.	Hyd-1- and Hyd-2-dependent H_2_ oxidation can support nitrate, fumarate, and oxygen respiration, thereby increasing the competitive fitness of *E. coli* in the inflamed gut.	Enhanced *E. coli* fitness and Enterobacteriaceae expansion.	Hughes et al., 2021
Biliary fibrosis	Preclinical—TAA-induced fibrosis in Sprague-Dawley rats	Category II	HRW	Changes in Clostridiaceae_1, *Ruminococcus*, *Turicibacter*, Coriobacteriales, *Actinomyces*, and Firmicutes bacterium.	Altered GM composition may contribute to host homeostasis and disease protection, although the functions of individual taxa and causal relationships remain unclear.	Reduced incidence and severity of TAA-induced cholangiofibrosis, decreased liver lesion area, delayed fibrosis progression, and improved survival-related outcomes.	Li et al., 2021
Radiation-induced gastrointestinal injury	Preclinical—total abdominal irradiation in C57BL/6 mice	Category II	HRW	Greater stability of GM structure. ↑Bacteroidetes and ↓Proteobacteria.	GM stabilization occurred in parallel with increased miR-1968-5p and reduced MyD88 expression. Microbiota mediation was not directly tested.	Improved survival, body weight, gastrointestinal function, and intestinal epithelial integrity.	Xiao et al., 2018
Chemotherapy -induced neuropathic pain	Preclinical—C57BL/6J mice	Category II	HRW	Altered microbial diversity and composition. ↑Firmicutes, *Lactobacillus*, Lactobacillaceae. ↓Bacteroidetes and *Bifidobacterium*.	GM remodeling and changes in LPS–TLR4-related indices were associated with reduced inflammation and oxidative stress.	Reduced mechanical hyperalgesia and neuropathic pain, with lower inflammatory and oxidative-stress markers.	Lian et al., 2021
Constipation	Preclinical—experimental constipation in Sprague-Dawley rats	Category II	HRW	Changes in *Romboutsia* and Oscillospiraceae_UCG-005, with altered β-leucine and traumatic acid. ↑Firmicutes. ↓Bacteroidetes and Actinobacteria.	A taxa-metabolite-SIRT1/Nrf2/HO-1 pathway has been proposed, but microbial taxa and metabolites have not been shown to be necessary or sufficient.	Improved intestinal motility and constipation-related outcomes, with reduced intestinal oxidative stress.	Chen et al., 2024a
NSAID-induced enteropathy	Preclinical—indomethacin-induced enteropathy in mice; FMT and cecal-supernatant experiments	Category III	HRW	No significant short-term change in GM diversity or composition. ↑Intestinal and cecal acetate, propionate, and butyrate.	FMT from HRW-treated donors reduced injury, and microbiota-depleted cecal supernatants increased macrophage IL-10. These findings support a contribution from microbial metabolic activity, particularly SCFAs.	Reduced small-intestinal ulceration, hyperpermeability, oxidative stress, and inflammatory cytokine expression.	Akita et al., 2023
Parkinson’s disease	Clinical observation—patients with PD	Category I	No direct exogenous H_2_ intervention	↓Putative H_2_-producing bacteria and estimated intestinal H_2_ production. H_2_-generating *Prevotella* was inversely associated with disease severity. Non-H_2_-producing lactic acid bacteria were increased in one report.	Reduced endogenous intestinal H_2_ production may be related to altered redox, inflammatory, and neuroprotective capacity. Evidence is observational.	Associations with PD presence or severity; no direct treatment outcome.	Suzuki et al., 2018; Mao et al., 2021
Parkinson’s disease	Preclinical—PD animal model	Category II	ERW	↑Butyrate-producing taxa and fecal butyrate, with improved intestinal barrier integrity.	Changes in intestinal redox conditions, butyrate metabolism, and barrier function may contribute to gut-liver-brain effects.	Favorable changes in intestinal, hepatic, and brain pathology.	Bordoni et al., 2019
Alzheimer’s disease	Preclinical—3×Tg-AD mice	Category II	HRW for 7 months	↑Lachnospiraceae and Ruminococcaceae. ↓*Helicobacter*.	Restoration of microbial diversity and taxa associated with carbohydrate, SCFA and amino-acid metabolism may support gut-brain metabolic and immune homeostasis.	Improved learning and memory; prevented synaptic loss and neuronal death; reduced Aβ plaques, tau hyperphosphorylation, neurofibrillary tangles, neuroinflammation and cerebral bioenergetic dysfunction	Lin et al., 2022
Alzheimer’s disease	Preclinical—AlCl_3_-induced zebrafish model	Category II	Nanobubble-generated HRW	Partially restored microbial diversity and community structure; reduced *Ralstonia*, *Staphylococcus aureus*, *Streptococcus* and *Corynebacterium*	Reduction of putative pathobionts may limit intestinal barrier disruption, endotoxin translocation and systemic or central inflammation.	Improved cognitive impairment and depression-like behavior; reduced Aβ deposition, neuroinflammation, oxidative stress, and neuronal injury.	He et al., 2025
Sepsis-associated encephalopathy	Preclinical—murine sepsis-associated encephalopathy	Category II	H_2_ inhalation or HRW	Shifts in GM composition, including ↑Akkermansia, with changes in serum and brain metabolites and associations with tryptophan metabolism.	GM and tryptophan-related metabolic pathways may contribute to the observed neuroprotective response.	Improved cognitive outcomes and survival, with reduced neuroinflammation.	Han et al., 2023
Methamphetamine addiction	Clinical—non-randomized controlled—Methamphetamine abusers	Category II	HRW or H_2_ inhalation for 3 months	Only H_2_ inhalation was accompanied by ↑microbial diversity and community shifts. *Bacteroides* abundance was inversely associated with symptom severity.	GM changes may contribute to neuropsychiatric effects through gut-brain signaling, but mediation was not established.	Lower anxiety and depressive symptom scores. Microbial effects differed between HRW and inhaled H_2_.	Wang et al., 2023
Opioid addiction—animal study	Preclinical—morphine-conditioned mice	Category II	H_2_ released from coral calcium hydride	Partial reversal of morphine-associated dysbiosis and changes in circulating SCFAs. ↑Bacteroidetes, *Lactobacillus*, *Alloprevotella*. ↓Firmicutes.	Changes in microbial community structure and SCFA metabolism may contribute to gut-brain effects.	Facilitated extinction of morphine-conditioned place preference and reduced morphine-primed reinstatement.	Xie et al., 2024
Opioid addiction—human study	Clinical—non-randomized controlled—participants with opioid addiction	Category II	H_2_ inhalation for 3 months	Shifts in microbial community structure without a clear increase in diversity. ↑Bacteroidetes and Actinobacteria. ↓Lachnospiraceae.	Coordinated changes in GM composition and metabolites may influence gut-brain-axis signaling.	Reduced anxiety and depression scores, with no significant change in opioid craving.	Xie et al., 2024
Asthma	Preclinical—asthmatic mice	Category II	HRW	↑*Bifidobacterium* and *Lactobacillus*/*Ligilactobacillus*. ↓*Parabacteroides*. ↑Indole-3-acetic acid.	A microbiota-tryptophan metabolite-AhR pathway has been proposed.	Reduced type 2 airway inflammation, inflammatory-cell infiltration, mucus secretion, and goblet-cell metaplasia, with improved epithelial integrity.	Li et al., 2024c
Chronic hypoxia-related multiorgan injury and bone loss	Preclinical—chronic hypoxia model	Category II	HRW and CCH	Partial reversal of hypoxia-associated dysbiosis, including restoration of reduced *Lactobacillus*.	A possible GM-systemic inflammation-gut-bone axis was proposed.	Reduced systemic inflammation, multiorgan injury, and bone loss.	Zhu et al., 2026
Radiation-induced oral mucositis	Preclinical—radiation-induced oral mucositis model	Category II	HRW	Partial normalization of radiation-associated changes, including reduced expansion of *Streptococcus* and *Helicobacter* and preservation of SCFA-producing taxa.	Preservation of intestinal barrier function and SCFA-associated microbial ecology may contribute to lower oral and intestinal inflammation.	Reduced oral and intestinal inflammation and improved intestinal barrier integrity.	Lan et al., 2025

Abbreviations: AD, Alzheimer’s disease; AhR, aryl hydrocarbon receptor; ALD, alcohol-associated liver disease; CCH, coral calcium hydride; DSS, dextran sulfate sodium; FMT, fecal microbiota transplantation; GM, gut microbiota; HRS, H2-rich saline; HRW, H2-rich water; ERW, electrolyzed reduced water; IFG, impaired fasting glucose; LPS, lipopolysaccharide; MASLD, metabolic dysfunction-associated steatotic liver disease; MyD88, myeloid differentiation factor 88; NF-κB, nuclear factor kappa-B; NSAID, nonsteroidal anti-inflammatory drug; PD, Parkinson’s disease; PPAR-γ, peroxisome proliferator-activated receptor-γ; SCFAs, short-chain fatty acids; SIRT1, silent information regulator 1; Nrf2, nuclear factor erythroid 2-related factor 2; HO-1, heme oxygenase-1; TAA, thioacetamide; TLR4, toll-like receptor 4; UC, ulcerative colitis.

• Category I: Studies of endogenous intestinal H2 metabolism or microbial H2 utilization without therapeutic administration of exogenous H_2_.

• Category II: Parallel GM and disease-related changes after exogenous H2 administration, without direct experimental testing of microbiota mediation.

• Category III: Intervention-supported evidence consistent with a microbiota-dependent contribution, based on antibiotic depletion, FMT, microbiota transfer, or related approaches. Such evidence strengthens support for microbiota involvement but does not, by itself, establish microbiota mediation.

For Category II studies, concurrent changes in GM, microbial metabolites, host signaling, and disease phenotypes are interpreted as associations and do not establish microbiota mediation. Direct effects of H2 cannot be excluded.

### 3.1 Metabolic diseases

Metabolic diseases are commonly accompanied by marked GM dysbiosis, which leads to chronic low-grade inflammation, intestinal barrier disruption, and metabolic imbalance (Wang Y. et al., 2025). H_2_ has shown promising potential in both the prevention and treatment of such diseases, with reported changes in metabolic, inflammatory, barrier-related, and microbial outcomes. In high-fat diet-fed mice, HRS can ameliorate dyslipidemia with, partial normalization of GM composition, improved intestinal mucosal pathology, and altered activities of key metabolic enzymes (Qiu et al., 2020). Randomized controlled clinical trials have further confirmed that long-term consumption of high-concentration HRW significantly improved body composition, lipid profiles, and glycemic indicators in patients with metabolic syndrome, while reducing oxidative stress and inflammation levels, all with favorable safety profiles (LeBaron et al., 2020) (Figure 3a).

**FIGURE 3:**
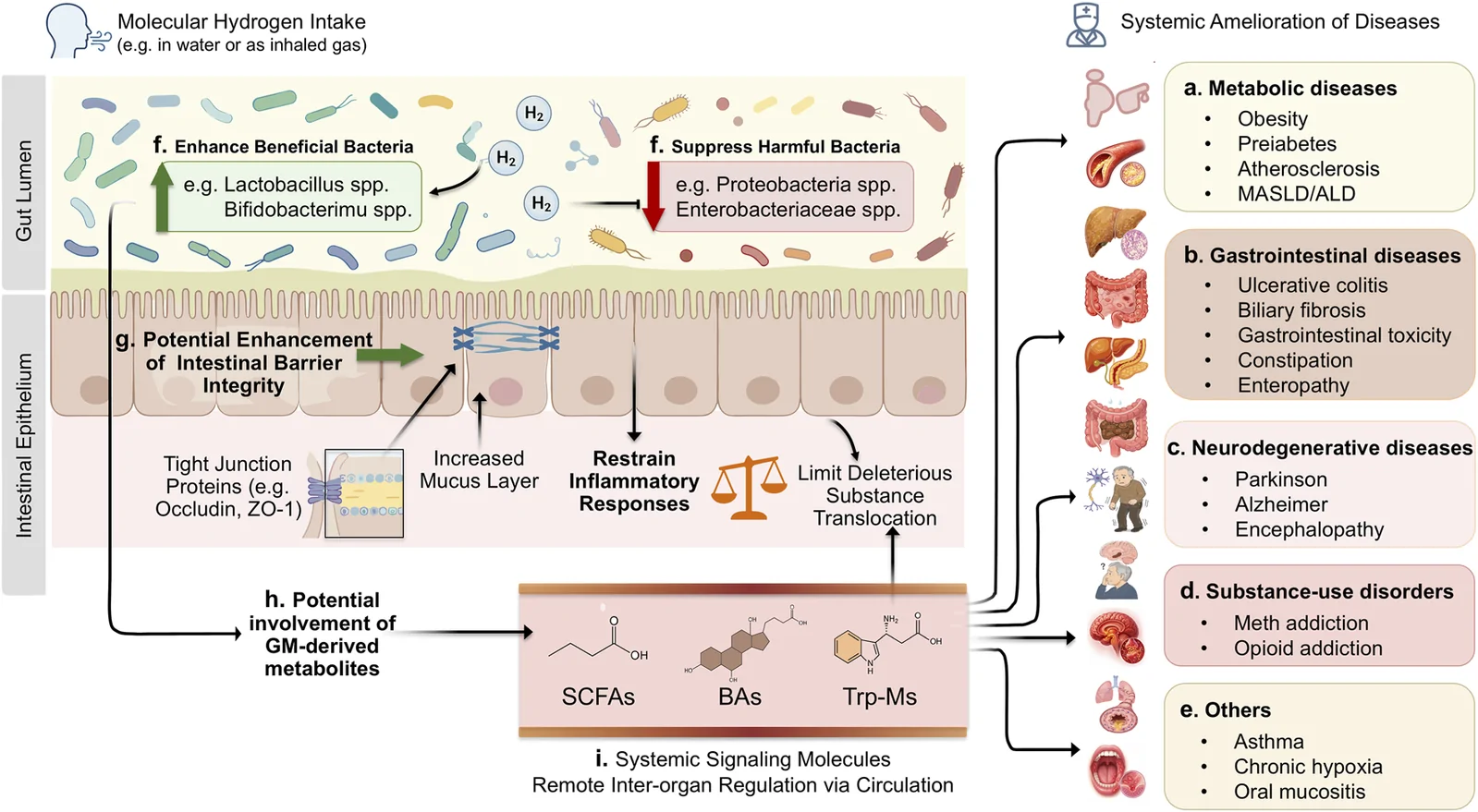
Proposed GM-associated mechanisms underlying disease amelioration by H_2_. H_2_ intake may contribute to the amelioration of **(a)** metabolic diseases, **(b)** gastrointestinal diseases, **(c)** neurodegenerative diseases, **(d)** substance-use disorders, and **(e)** other conditions. H_2_ has been associated with enrichment of putatively beneficial bacteria and reduction of potentially harmful taxa **(f)**. These changes may contribute to enhanced intestinal barrier integrity, increased mucus production, attenuated inflammatory responses, and reduced translocation of deleterious substances **(g)**. H_2_-associated alterations in the GM may also affect microbiota-derived metabolites, including SCFAs, BAs, and tryptophan metabolites **(h)**, which may enter the circulation and act as systemic signaling molecules involved in inter-organ communication **(i)**. Abbreviations: GM, gut microbiota; SCFAs, short-chain fatty acids; BAs, bile acids; Trp-Ms, tryptophan metabolites; Meth, methamphetamine; MASLD, metabolic dysfunction-associated steatotic liver disease; ALD, alcohol-associated liver disease.

#### 3.1.1 Obesity and prediabetes

In recent years, the possible involvement of GM in the onset and progression of obesity has attracted increasing attention (Gomes et al., 2018). An increased Firmicutes/Bacteroidetes ratio and greater oxidative stress have been reported in some obesity-associated microbial profiles (Zhang S. et al., 2025). In obese mouse models, H_2_ administration attenuated hepatic lipid accumulation and oxidative stress and was accompanied by changes in the GM. Furthermore, H_2_ treatment was associated with partial normalization of GM composition and improvements in metabolic abnormalities (Qiu et al., 2020). Notably, sustained-release H_2_ nanocapsules were also reported to increase the intestinal abundance of *Akkermansia muciniphila*, improve gut microbial homeostasis, and consequently alleviate multiple metabolic abnormalities in mice, including early MAFLD, obesity, and diabetes (Jin et al., 2021). Collectively, these preclinical findings suggest that improvements in obesity-related metabolic phenotypes are accompanied by alterations in the GM. Consistent with these findings, GM dysbiosis is widely observed in patients with diabetes and may be associated with disease progression (Zhang et al., 2013). In a randomized, double-blind, placebo-controlled trial involving 73 individuals with impaired fasting glucose (IFG), Liang et al. reported that 8 weeks of HRW intervention significantly reduced fasting blood glucose and visceral fat area, while also markedly altered gut microbial composition. Notably, changes in microbial taxa were associated with several metabolites (Liang et al., 2023). However, because the microbiota was not directly manipulated in these studies, the evidence for GM mediation remains primarily associative.

#### 3.1.2 Atherosclerosis

Recent preclinical evidence provides comparatively stronger support for a microbiota-dependent component of the vascular benefits of H_2_. Meng et al. demonstrated that HRW consumption significantly reduced atherosclerotic lesion formation and improved plaque stability in ApoE^−/−^ mice. This protective effect was associated with GM remodeling, increased SCFA production, and reduced pro-inflammatory M1 macrophage polarization. Importantly, depletion of the GM with antibiotics abolished the anti-atherosclerotic effect of HRW, whereas FMT from HRW-treated donor mice partially reproduced these effects in recipient mice. These intervention-based findings provide comparatively strong support for microbiota involvement in the observed response, although they do not establish definitive microbiota-mediated causality (Meng et al., 2026).

#### 3.1.3 MASLD and ALD

Both metabolic dysfunction-associated steatotic liver disease (MASLD) and alcohol-associated liver disease (ALD) are closely linked to GM dysbiosis. Animal studies have shown that H_2_ inhalation may increase the abundance of *A*. *muciniphila*, restore intestinal barrier integrity, and alleviate metabolic dysfunction and liver injury in high-fat diet-induced MASLD (Xue et al., 2023; Wang et al., 2024). In ALD mice models, H_2_ attenuated liver injury in association with changes in GM composition. Moreover, FMT experiments provide intervention-supported evidence for GM involvement in the hepatoprotective effects of H_2_ (Liu et al., 2023).

### 3.2 Gastrointestinal diseases

Preclinical studies have demonstrated that modulation of gut microbial composition can effectively alleviate a range of gastrointestinal disorders, highlighting the GM as a key therapeutic target. Notably, exogenous H_2_ has also produced favorable outcomes in several experimental digestive disorders, often alongside changes in the GM-intestinal barrier axis (Li J. et al., 2024) (Figure 3b).

#### 3.2.1 Ulcerative colitis

Ulcerative colitis (UC) is an immune-mediated chronic inflammatory disorder that primarily affects the rectum and colon (Li J. et al., 2024). In dextran sulfate sodium (DSS)-induced colitis models, HRW reduced disease severity and improved intestinal barrier integrity, accompanied by alteration in GM modulation. HRW has been shown to suppress the overgrowth of pathogenic bacteria such as *Enterococcus spp.* and *Clostridium perfringens*, thereby partially restoring microbial homeostasis (Song et al., 2022). Additionally, HRS administration was also associated with increased abundance of several SCFA-producing bacteria, including *Eubacterium ventriosum*, *Roseburia hominis*, and *Roseburia faecis*, together with changes in mucosa-associated *Akkermansia muciniphila* and *Ruminococcus gnavus*, and improved mucus and barrier integrity (Ge et al., 2022). Separately, UC patients and DSS-treated mice showed increased abundance of genes encoding predicted H_2_-uptake hydrogenases, while Hyd-1- and Hyd-2-dependent H_2_ oxidation enhanced the competitive expansion of commensal *Escherichia coli* in the inflamed gut. This finding reflects endogenous microbial H_2_ metabolism rather than a therapeutic H_2_ response (Hughes et al., 2021). HRW was also broadly comparable to sulfasalazine in experimental colitis, although combined treatment showed only a general tendency toward greater efficacy (LeBaron et al., 2021). Although these preclinical findings support H_2_ as a promising adjunctive strategy for UC, the underlying microbiota-associated metabolic networks and host-microbiota interactions remain to be elucidated (Hughes et al., 2021; Ge et al., 2022; Higashimura et al., 2018).

#### 3.2.2 Biliary fibrosis

In a thioacetamide (TAA)-induced rat model of cholangiofibrosis, long-term HRW administration improved general condition and reduced the incidence, number, and histological extent of biliary lesions. HRW was also associated with alterations in GM composition, including changes in *Clostridiaceae_1*, *Ruminococcus*, *Turicibacter*, *Coriobacteriales*, *Actinobacteria*, and uncultured *Firmicutes* bacteria. In a separate intervention experiment, HRW administered after withdrawal of TAA reduced the subsequent progression of cholangiofibrosis and liver lesion burden (Li et al., 2021). These findings provide primarily associative evidence for GM involvement.

#### 3.2.3 Radiotherapy-induced gastrointestinal injury

Radiotherapy is a major treatment modality for malignant tumors, but it is frequently associated with gastrointestinal toxicity (Chen et al., 2021). Emerging evidence suggests that HRW mitigates radiation-induced gastrointestinal injury, at least in part through modulation of the GM. In mice subjected to total abdominal irradiation, oral administration of HRW significantly improved survival, body weight, gastrointestinal function, and intestinal epithelium integrity, accompanied by greater stability of the gut microbial structure (Xiao et al., 2018). Similarly, HRW has been shown to alleviate oxaliplatin-induced mechanical hyperalgesia and chemotherapy-induced neuropathic pain while altering gut microbial diversity and composition (Lian et al., 2021). These preclinical findings suggest that H_2_ may confer systemic protection through microbiota-associated mechanisms.

#### 3.2.4 Constipation

In experimental constipation models, HRW alleviated constipation symptoms and intestinal oxidative stress. These effects were accompanied by changes in potentially beneficial taxa, including *Romboutsia* and *Oscillospiraceae_UCG-005*, and microbial metabolites such as β-leucine and traumatic acid (Chen K. D. et al., 2024). However, the current evidence remains hypothetical unless the relevant metabolites and microbial taxa are shown to be necessary or sufficient for the effect.

#### 3.2.5 NSAID-induced enteropathy

In a murine model of indomethacin-induced enteropathy, HRW alleviated small-intestinal ulceration, mucosal hyperpermeability, oxidative stress, and inflammatory cytokine expression. Although short-term HRW administration did not significantly alter gut microbial diversity or composition, it was associated with increased intestinal and cecal levels of acetate, propionate, and butyrate. Moreover, FMT from HRW-treated mice reduced intestinal injury, and altered microbial community composition, suggesting that the protective effects of H_2_ may involve GM-related functional changes. Notably, the protection reproduced by FMT provides intervention-supported evidence for microbiota-associated involvement, although it does not establish that specific microbial alterations mediate the response to H_2_ (Akita et al., 2023).

### 3.3 Neurodegenerative diseases

Both Parkinson’s disease (PD) and Alzheimer’s disease (AD) are closely associated with gut dysbiosis, barrier disruption, and chronic neuroinflammation (Czaj et al., 2026). Given that the GM can metabolize exogenous H_2_ and influence microbiota-host metabolic interface (Mukhopadhya and Louis, 2025), a role for the GM in H_2_-related neuroprotection has been proposed (Hu et al., 2025) (Figure 3c). However, current evidence remains largely inferential, as the microbiota or specific microbial metabolites have rarely been directly manipulated to establish their mediating roles in neurological outcome. The potential microbiota–gut–brain mechanisms are discussed in Section 4.

#### 3.3.1 Parkinson’s disease

Patients with PD exhibit gut microbial alterations that may provide a biological rationale for H_2_-based intervention, including reduced abundance of putative H_2_-producing bacteria, lower estimated intestinal H_2_ production, and an inverse association between H_2_-generating *Prevotella* and disease severity (Suzuki et al., 2018; Mao et al., 2021; Ostojic, 2018). In a PD animal model, HRW was associated with improved intestinal barrier integrity, enrichment of butyrate-producing taxa, and increased fecal butyrate levels, together with favorable changes in gut, liver, and brain pathology (Bordoni et al., 2019). Clinical evidence for HRW in PD remains inconclusive. A small pilot trial reported improved total UPDRS scores (Yoritaka et al., 2013), whereas a larger multicenter trial found no significant benefit (Yoritaka et al., 2018). Thus, gut dysbiosis and reduced endogenous H_2_ production may support H_2_ intervention in PD, while the contribution of GM modulation to its therapeutic effects warrants further investigation in humans.

#### 3.3.2 Alzheimer’s disease

Alterations in the GM have been reported in Alzheimer’s disease (AD), although the taxonomic patterns vary among populations. In a Puerto Rican case–control cohort, overall microbial diversity did not differ significantly between patients with AD and controls, whereas *Faecalibacterium* and *Bacteroides* were relatively enriched in controls. APOE ε4 carriers also showed lower abundance of butyrate-producing bacteria and enrichment of potentially pro-inflammatory genera, including *Eggerthella* and *Lachnoclostridium* (Sepúlveda-Rivera et al., 2025). These observations indicate an association between AD and gut microbial dysbiosis. Direct evidence linking H_2_, GM, and AD remains limited to preclinical studies. In 3×Tg-AD mice, 7 months of HRW reduced synaptic and neuronal loss, Aβ accumulation, tau hyperphosphorylation, accompanied by alterations in taxa such as *Lachnospiraceae_NK4A136_group*, *Ruminococcaceae_UCG-014*, and *Helicobacter* (Lin et al., 2022). In an AlCl_3_-induced zebrafish AD-like model, HRW improved behavioral deficits and reduced Aβ deposition, while partially restoring gut microbial composition, including decreases in *Ralstonia*, *Staphylococcus*, *Streptococcus*, and *Corynebacterium* (He et al., 2025). Moreover, by inhibiting histone deacetylase activity and upregulating neurogenesis-related genes such as *Bdnf*, H_2_ may promote neural stem cell proliferation (Kostiuchenko et al., 2024). Overall, these studies demonstrate parallel neurological and microbial changes, but do not establish that GM alterations mediate the neuroprotective effects of H_2_.

#### 3.3.3 Sepsis-associated encephalopathy

In mice with sepsis-associated encephalopathy, H_2_ inhalation or HRW was associated with better cognitive outcomes and improved survival, alongside shifts in gut microbial composition and changes in serum and brain metabolites, including enrichment of *Akkermansia* and associations between specific bacterial taxa and tryptophan metabolism. Together, these findings demonstrate parallel changes in neurological outcomes, GM composition, and microbial metabolites, but do not establish microbiota-mediated causality (Han et al., 2023).

### 3.4 Substance-use disorders

#### 3.4.1 Methamphetamine addiction

In female individuals undergoing detoxification from methamphetamine use, 3 months of H_2_ administration, either as HRW or inhaled gas, was associated with lower anxiety and depressive symptom scores. Notably, only H_2_ inhalation was accompanied by increased gut microbial diversity and shifts in microbial community structure, while the abundance of *Bacteroides* was inversely associated with symptom severity. These findings support an association, but do not establish that the microbial changes mediate the clinical effects of H_2_ (Wang et al., 2023).

#### 3.4.2 Opioid addiction

Using complementary animal and human studies, Xie et al. found that H_2_ administration was associated with improvements in opioid addiction-related outcomes and concurrent alterations in the GM. In morphine-conditioned mice, H_2_ released from coral calcium hydride facilitated behavioral extinction and reduced reinstatement, while partially reversing morphine-associated dysbiosis and modifying circulating SCFAs. In the human cohort, 3 months of H_2_ inhalation was associated with lower anxiety and depression scores and shifts in microbial community structure, although microbial diversity did not clearly increase. These findings support an association between GM-related changes and the behavioral or clinical effects of H_2_, but do not establish microbiota-mediated causality on opioid addiction (Xie et al., 2024) (Figure 3d).

### 3.5 Other pathological conditions

Beyond the diseases discussed above, growing evidence suggests that microbiota-related changes may also accompany the protective effects of H_2_ in other pathological settings (Figure 3e). In asthmatic mice, HRW was associated with enrichment of *Bifidobacterium* and *Lactobacillus*, enhanced production of the tryptophan metabolite indole-3-acetic acid, and reduced type-2 airway inflammation (Li L. et al., 2024). Under chronic hypoxia, H_2_ intervention partially reversed gut microbial dysbiosis, including the reduction in *Lactobacillus*, and was accompanied by alleviation of systemic inflammation, multi-organ injury, and bone loss, suggesting a possible involvement of the gut-bone axis (Zhu et al., 2026). Similarly, in radiation-induced oral mucositis, HRW preserved intestinal barrier integrity and partially normalized radiation-associated microbial alterations, including expansion of *Streptococcus* and *Helicobacter* and depletion of SCFA-producing taxa, changes that were associated with reduced oral and intestinal inflammation (Lan et al., 2025).

Emerging clinical evidence also suggests potential benefits of H_2_ in myalgic encephalomyelitis/chronic fatigue syndrome (ME/CFS) and Long COVID. Preliminary clinical evidence suggests potential benefits of HRW for fatigue-related outcomes in these conditions, although the evidence remains limited (Friedberg et al., 2025; Tan et al., 2024). GM dysbiosis has also been independently reported in both conditions, including reduced butyrate-producing capacity in ME/CFS and persistent microbial alterations associated with post-acute COVID-19 symptoms (Guo et al., 2023; Liu Q. et al., 2022). However, current H_2_ intervention studies have not assessed the GM, and whether microbiota modulation contributes to the potential effects of H_2_ remains unknown (Friedberg and LeBaron, 2026).

## 4 Proposed mechanisms underlying the gut microbiota-associated effects of molecular hydrogen

In this mechanistic framework, putative direct effects refer to host responses to H_2_ that occur independently of demonstrated GM alterations, whereas indirect microbiota-associated effects involve host responses linked to changes in microbial composition or metabolites.

### 4.1 Modulation of gut microbiota composition and ecological balance

The structural balance of GM is fundamental to its physiological functions, and dysbiosis is typically characterized by depletion of beneficial microorganisms together with expansion of harmful taxa (Figure 3f). Regulation of microbial composition, either directly or indirectly, has been proposed to contribute to the biological effects associated with H_2_. As a small molecule with high lipid solubility and rapid diffusibility, H_2_ can readily penetrate biological membranes and enter the intestinal microbial niche, where it may be selectively utilized by specific commensals, including sulfate-reducing bacteria and methanogens (Birg et al., 2023), thereby influencing the competitive balance between H_2_-producing and H_2_-consuming microorganisms. In parallel, H_2_ may scavenge reactive oxygen species (ROS) in the gut and lower the intestinal oxidation-reduction potential (Li J. et al., 2024), thus creating a more favorable environment for the growth of beneficial anaerobes. Harmful bacteria generate toxic metabolites during metabolism, including endotoxins such as LPS, as well as indole and sulfide. Once absorbed into circulation, these products can promote chronic low-grade inflammation and tissue injury. Available evidence further suggests that H_2_ treatment may suppress the growth and proliferation of harmful bacteria, potentially through the downregulation of key metabolic genes, thereby reducing the production of toxic metabolites. Both H_2_ inhalation and HRW intake have been associated with increased relative abundance of beneficial taxa, such as *Lactobacillus* and *Bifidobacterium*, together with reduced abundance or overgrowth of potentially pathogenic bacteria, including members of Enterobacteriaceae and the *Escherichia-Shigella* group (Zang et al., 2024; Shi et al., 2025; Li L. et al., 2024). In addition, H_2_ may strengthen antagonistic interactions between beneficial and harmful microorganisms, thereby further contributing to the maintenance of gut microbial homeostasis (Han et al., 2023).

### 4.2 Potential enhancement of intestinal barrier integrity

The intestinal mucosal barrier constitutes a primary line of defense against the invasion of exogenous pathogens and endotoxins. Barrier disruption caused by gut dysbiosis is a major driver of endotoxin translocation and systemic inflammation. H_2_ may contribute to barrier protection by promoting the proliferation of beneficial bacteria and enhancing the production of SCFAs (Figure 3g). Among these metabolites, butyrate serves as a major energy source for intestinal epithelial cells, promotes their proliferation and differentiation, and upregulates the expression of tight junction proteins, including occludin, ZO-1, and claudins, thereby reducing intestinal permeability (Ge et al., 2022). At the same time, host-derived defensins and bacterially produced bacteriocins can inhibit the adhesion and invasion of harmful microorganisms, further limiting mucosal injury (Dobson et al., 2012). In the context of inflammatory regulation, H_2_ treatment was associated with reduced activation of TLR4/nuclear factor kappa-B (NF-κB) signaling pathway, together with lower abundance of harmful bacteria, reduced LPS generation, and decreased release of pro-inflammatory cytokines such as tumor necrosis factor-α (TNF-α), interleukin-6 (IL-6), and IL-1β. In parallel, SCFAs activate G protein-coupled receptor 43 (GPR43) and GPR41 signaling, inhibit NF-κB activation, reduce pro-inflammatory mediator expression, and promote the production of anti-inflammatory cytokines such as IL-10, thus helping maintain immune homeostasis (Liao et al., 2022; Huang et al., 2020). These SCFA-related pathways therefore represent a plausible indirect, microbiota-associated mechanism linking H_2_-related microbial changes to intestinal immune regulation. In parallel with these microbiota-associated pathways, H_2_ may also exert direct effects on the intestinal redox environment, including ROS scavenging, thereby potentially attenuating mucosal oxidative damage and inflammatory responses.

### 4.3 Potential involvement of gut microbiota-derived metabolites

Microbiota-derived metabolites are key mediators through which GM influences host health. H_2_-induced changes in GM composition have been associated with altered levels of several potentially beneficial microbial metabolites (Ge et al., 2022) (Figure 3h). H_2_ may also modulate other microbial secondary metabolic pathways. For example, H_2_ promotes bacterial 21-dehydroxylation of host corticoids into progestins and neurosteroids (McCurry et al., 2024).

SCFAs are major end products of dietary fiber fermentation by the GM. Among them, butyrate serves as the principal energy source for intestinal epithelial cells and enhances mucosal barrier integrity through two complementary mechanisms. It promotes mucus layer thickening by regulating mucin-related gene expression in goblet cells and reinforces tight junction architecture through activation of the AMP-activated protein kinase pathway (Li et al., 2025). Propionate contributes to glucose homeostasis by suppressing hepatic gluconeogenesis, whereas acetate participates in the regulation of lipogenesis and energy balance (Mukhopadhya and Louis, 2025). Accumulating evidence indicates that H_2_ may increase overall SCFAs levels by enriching SCFA-producing bacteria and has been associated with upregulated expression of key microbial genes involved in SCFAs biosynthesis, including those encoding acetate kinase and butyryl-CoA transferase (Mukhopadhya and Louis, 2025; Ge et al., 2022). In addition to their metabolic functions, SCFAs exert marked anti-inflammatory effects. Butyrate suppresses the synthesis and release of pro-inflammatory mediators in the colonic lamina propria, thereby reducing the risk of intestinal inflammation (Li et al., 2025), whereas acetate and propionate exert anti-inflammatory effects through different signaling pathways (Mukhopadhya and Louis, 2025). H_2_ may further influence secondary microbial metabolism, including bile acid transformation, by altering H_2_-sensitive microbial redox processes. Such changes may modify the profile of secondary bile acid metabolites, although their net physiological consequences remain context dependent (Hylemon et al., 2018). Accordingly, H_2_-associated enrichment of SCFA-producing bacteria may be accompanied by increased production of key metabolites such as butyrate and propionate. Once released into circulation, these metabolites may act as systemic signaling molecules to modulate distant organs, including the liver, adipose tissue, and central nervous system, thereby contributing to inter-organ regulation (Mann et al., 2024). Nevertheless, direct evidence demonstrating that these metabolites mediate the systemic effects associated with H_2_ remains limited.

### 4.4 Other proposed regulatory mechanisms

Beyond SCFAs, H_2_ may also be associated with disease-related changes through potential effects on BAs and tryptophan metabolism (Figure 3i). The GM converts primary BAs synthesized in the liver into secondary BAs, which in turn regulate host metabolism and immune responses through signaling pathways mediated by the farnesoid X receptor and the G protein-coupled bile acid receptors (Lee et al., 2024). H_2_ may be associated with the increased generation of anti-inflammatory and metabolically beneficial secondary BAs, and reduced production of potentially toxic species such as deoxycholic acid. These changes may involve alterations in the abundance of BA-transforming bacteria, including *Bacteroides* and *Clostridium* (Hylemon et al., 2018; Zeng et al., 2026). Through these BA-dependent signaling pathways, H_2_-associated changes in BA metabolism may contribute to improvements in host metabolic and inflammatory status and may potentially attenuate the progression of metabolic syndrome. Additionally, H_2_ may optimize the profile of microbiota-derived tryptophan metabolites, thereby enhancing AhR signaling and contributing to intestinal immune homeostasis and barrier function. For example, in asthma models, H_2_ has been shown to exert anti-inflammatory effects by increasing serum levels of IAA (Li L. et al., 2024).

Overall, the proposed microbiota-mediated effects of H_2_ may involve a multilayered and interrelated regulatory network. H_2_ may influence gut microbial homeostasis by enriching beneficial taxa and suppressing the expansion of harmful microorganisms. Such microbial changes may be accompanied by improved intestinal barrier function, reduced translocation of deleterious substances, attenuated inflammatory responses, and preservation of intestinal homeostasis. In parallel, microbiota-derived metabolites, including SCFAs, BAs, and tryptophan metabolites, may act as systemic signaling molecules and have been proposed to participate in remote inter-organ regulation. These mechanisms are closely interconnected and mutually reinforcing, collectively forming the plausible framework for explaining how GM-related changes may contribute to the disease-ameliorating effects associated with H_2_ (Figure 3). Nevertheless, most available studies demonstrate parallel changes in microbial composition, metabolite profiles, inflammatory markers, and disease phenotypes rather than direct microbiota-mediated causality. Where antibiotic depletion or FMT modifies or transfers the H_2_-associated phenotype, these findings provide stronger, intervention-supported evidence for microbiota involvement, but still do not establish that specific microbial alterations mediate the therapeutic effect. Further studies are needed to determine whether GM alterations are necessary or sufficient for the observed effects of H_2_.

## 5 Administration routes, exposure dynamics, and safety considerations of exogenous H_2_

The route of H_2_ administration influences its spatial and temporal exposure profiles, the dose reaching target tissues, and the feasibility of repeated treatment, while potentially affecting the relative roles of luminal and systemic pathways in GM modulation (Table 2).

**TABLE 2:** Comparative microbiota-related characteristics and potential clinical contexts of different exogenous H_2_ administration routes.

Route	Predominant exposure profile	Reported temporal pattern	Reported research/application contexts	Microbiota-related effects and proposed mechanisms	Main advantages/limitations	Representative refs.
H_2_-rich water (HRW; oral)	Direct gastrointestinal luminal exposure to dissolved H_2_, followed by portal and systemic absorption.	Intermittent luminal exposure after each intake; microbiota-related changes are generally reported after repeated administration over days to weeks.	Clinical: healthy adults and impaired fasting glucose. Experimental/preclinical: metabolic disease, atherosclerosis, colitis, hepatobiliary disease, radiation- or NSAID-related gastrointestinal injury, constipation, neurodegenerative disease, and asthma.	Provides the most direct opportunity for luminal microbiota modulation. Reported effects include enrichment of beneficial or H_2_-producing taxa, regulation of SCFA and tryptophan metabolism, improved intestinal barrier function, and attenuation of oxidative and inflammatory responses. Causal GM mediation is shown only in selected models.	A: non-invasive, convenient, and suitable for repeated administration. L: rapid H_2_ loss, variable delivered dose, and dependence on storage conditions and gastrointestinal transit.	Liang et al., 2023; Akita et al., 2023; Meng et al., 2026
H_2_ inhalation	Rapid pulmonary uptake with controllable systemic exposure, but without direct delivery of H_2_ to the intestinal lumen.	Rapid systemic exposure during inhalation followed by a rapid decline after exposure ceases. GM responses are delayed and likely host mediated.	Clinical: ulcerative colitis, methamphetamine addiction, and opioid addiction. Experimental/preclinical: MASLD, alcohol-associated liver disease, and sepsis-associated encephalopathy. These studies do not establish indications.	May indirectly modulate the GM through systemic redox regulation, attenuation of inflammatory and immune signaling, restoration of intestinal barrier function, and regulation of microbial metabolites.	A: controllable concentration and exposure duration, with rapid systemic delivery. L: equipment and supervision requirements, fire-safety considerations, and limited direct intestinal exposure.	Sano et al., 2020; Liu et al., 2023; Wang et al., 2023; Maruyama et al., 2025
Rectal H_2_ delivery	Localized H_2_ delivery to the rectum and distal colon, with limited and potentially variable systemic absorption.	Exposure depends on formulation, volume, retention time, stool burden, and mucosal integrity. Local and broader GM kinetics remain poorly defined.	Experimental/preclinical local administration in models of distal colonic inflammation or mucosal injury. No established clinical indication has been demonstrated.	May directly affect the distal colonic mucosa-microbiota interface by altering the local oxidative and inflammatory microenvironment. Effects on the broader intestinal microbial community remain insufficiently established.	A: avoids upper gastrointestinal H_2_ loss and permits targeted distal delivery. L: poor acceptability, procedural variability, restricted anatomical coverage, and insufficient safety and dose data.	Liu T. et al., 2025
H_2_-rich saline (HRS; IV or IP)	Parenteral, extra-luminal H_2_ exposure after IV infusion or IP injection, without direct intestinal luminal delivery. IV and IP exposure profiles are route dependent.	IV H_2_ is largely lost during pulmonary passage, yielding minimal arterial exposure. After IP injection, venous H_2_ rises but arterial levels remain much lower. GM effects are mainly reported after repeated animal dosing.	Experimental/preclinical: IP HRS has been studied in DSS colitis and intestinal ischemia-reperfusion injury, the latter without direct GM profiling. IV evidence is pharmacokinetic only. No established clinical indication.	In animal models, IP HRS may influence the GM by attenuating intestinal inflammation, preserving barrier integrity, and increasing the abundance of SCFA-producing bacteria.	A: controlled dosing in preclinical studies. L: invasive administration, limited clinical translatability, and requirements for standardized preparation, sterility, and dosing.	Jiang et al., 2020 (IP) Ge et al., 2022 (IP) Shibuya et al., 2025 (IV)

Abbreviations: A, advantages; GM, gut microbiota; HRS, H2-rich saline; HRW, H2-rich water; IP, intraperitoneal; IV, intravenous; L, limitations; MASLD, metabolic dysfunction-associated steatotic liver disease; NSAID, nonsteroidal anti-inflammatory drug; SCFA, short-chain fatty acid.

### 5.1 Pharmacological characteristics and potential clinical contexts of H_2_ administration routes

HRW provides direct exposure of the gastrointestinal lumen to dissolved H_2_, followed by absorption into the portal circulation and systemic elimination. Its non-invasive nature and ease of repeated administration make HRW suitable for long-term outpatient interventions, particularly in chronic gastrointestinal and metabolic disorders. Oral administration currently has the relatively strongest direct human evidence for GM modulation, although the evidence remains limited. In healthy adults, consumption of H_2_-dissolved alkaline electrolyzed water for 2 weeks increased the relative abundance of *Bifidobacterium* and shifted stool consistency toward the normal range (Tanaka et al., 2021), while an 8-week randomized trial in patients with impaired fasting glucose reported partial correction of gut microbial dysbiosis (Liang et al., 2023). Animal studies using H_2_-sensitive microelectrodes have further shown that HRW increased intestinal H_2_ availability and was accompanied by changes in microbial composition and SCFAs production (Meng et al., 2026). However, the nominal H_2_ concentration in water does not directly represent the dose reaching the distal intestine because exposure is influenced by the concentration at consumption, drinking pattern, gastric emptying, mucosal absorption, and intestinal transit. Comparable segment-resolved and time-resolved measurements in humans remain unavailable.

H_2_ inhalation enables rapid pulmonary uptake and relatively direct control of the inspired concentration and treatment duration, although tissue exposure varies among organs. In pigs, arterial H_2_ concentrations increased rapidly during inhalation and declined soon after exposure ceased (Sano et al., 2020). Real-time measurements in mice also revealed organ-specific concentrations and equilibration times (Zhu et al., 2022). Because inhalation does not primarily expose the intestinal lumen, its effects on the GM may involve host-mediated changes in inflammatory and immune signaling and intestinal barrier integrity. In a randomized trial of patients with ulcerative colitis, H_2_ inhalation increased microbial diversity but did not clearly improve clinical disease activity (Maruyama et al., 2025). Inhalation offers a means of rapid and supervised systemic exposure but requires specialized equipment and controlled delivery.

Rectal administration bypasses the upper gastrointestinal tract and enables localized H_2_ delivery to the rectum and distal colon, making it a potential approach for experimental treatment of distal colitis and other localized mucosal inflammatory disorders. Enema-based H_2_-generating systems, such as chitosan-encapsulated H_2_ microbubbles, have prolonged local H_2_ release and attenuated oxidative stress and inflammation in murine colitis models (Liu T. et al., 2025). However, this route provides limited exposure to the proximal colon and small intestine, and its efficacy may be affected by formulation stability, administration volume, retention time, stool burden, and mucosal integrity. Limited patient acceptability, the absence of human dose-ranging, and microbiota studies further constrain its clinical translation.

HRS provides systemic H_2_ exposure without direct delivery to the intestinal lumen. Its effects on the GM are therefore likely to be indirect, involving reduced systemic inflammation, improved intestinal microcirculation and barrier integrity, and decreased bacterial or endotoxin translocation. In animal models, HRS attenuated DSS-induced colitis, increased SCFA-producing taxa, and preserved epithelial tight junctions in intestinal ischemia-reperfusion injury (Jiang et al., 2020; Ge et al., 2022). However, pharmacokinetic data from pigs indicates that most intravenously infused H_2_ is lost during pulmonary passage, leaving the effective peripheral tissue dose uncertain (Shibuya et al., 2025). HRS therefore remains primarily an experimental option when oral administration is impractical or systemic delivery is intended. Its clinical translation is limited by the lack of validated dosing regimens and standardized procedures for preparation, storage, sterility, concentration verification, and infusion.

Overall, HRW appears most feasible for repeated ambulatory interventions, whereas inhalation provides more controllable systemic exposure. Rectal delivery may provide localized distal-colonic exposure, and HRS may be relevant when oral treatment is impractical. However, the latter two approaches remain predominantly experimental, and these route-specific considerations should not be interpreted as established clinical recommendations.

### 5.2 Temporal dynamics of H_2_ exposure and gut microbiota modulation

H_2_ exposure is transient across administration routes, whereas microbiota responses are likely to develop over a longer timescale. Oral administration and inhalation increase portal or arterial H_2_ within minutes, whereas intravenously administered HRS is markedly reduced during pulmonary passage (Ichihara et al., 2021; Shibuya et al., 2025). Rectal exposure kinetics remain poorly characterized and likely depend on formulation stability and retention time. In contrast, human microbiota changes have been assessed only after repeated interventions. Alterations were reported after 2 weeks of H_2_-dissolved alkaline electrolyzed water and 8 weeks of HRW, but sampling was largely limited to baseline and study completion (Tanaka et al., 2021; Liang et al., 2023). The onset, peak, persistence, and reversibility of route-specific microbiota responses therefore remain unknown. Future studies should combine serial microbiome profiling with measurements of luminal, blood, or exhaled H_2_ during treatment and after withdrawal.

### 5.3 Dose regimens and safety considerations

No universally accepted therapeutic dose range, maximum tolerated exposure, or microbiota-specific safety threshold has been established for H_2_. Clinical studies have evaluated markedly different regimens, including 0.5 L/day of HRW containing 15 ppm H_2_ for 6 months (Zanini et al., 2021), more than 5.5 mmol H_2_/day for 24 weeks (LeBaron et al., 2020), and inhalation of 2.4% H_2_ for up to 72 h (Cole et al., 2021). These regimens describe exposures that have been investigated under specific conditions, but they do not define equivalent doses or universal safety boundaries. Concentrations expressed as ppm in water, mmol per day, percentages in inhaled gas, or concentrations in saline cannot be directly compared because each route produces different systemic exposure profiles. Moreover, a monotonic concentration–response relationship has not been established, and higher administered concentrations should not be assumed to produce greater microbiota or clinical effects.

Given the absence of standardized dose recommendations and validated safety thresholds, the potential risks associated with higher H_2_ exposure warrant careful consideration. The most clearly established hazards are related to the handling and delivery of gaseous H_2_ rather than to its demonstrated systemic toxicity. H_2_ is flammable at approximately 4–75 vol% in air. Therefore, high-concentration inhalation requires controlled gas mixing, adequate ventilation, leak detection, avoidance of ignition sources, and certified delivery devices (Sano et al., 2018). Biologically, H_2_ may exert context-dependent, potentially double-edged effects on the intestinal microbiota. Dysregulated intestinal H_2_ metabolism may be harmful in both directions. Excessive H_2_ production is associated with bloating, abdominal discomfort, and diarrhea (Šuran et al., 2026; Barlow and Pimentel, 2025), whereas excessive H_2_ consumption by methanogens and sulfate-reducing bacteria may shift microbial metabolism toward CH_4_ and H_2_S. CH_4_ is linked to delayed intestinal transit and constipation, while excessive H_2_S may impair mucus integrity and promote epithelial dysfunction and inflammation (Triantafyllou et al., 2014; Talamantes et al., 2024; Huang et al., 2024). Thus, altered H_2_ availability may influence microbial competition and metabolic outputs in ways that are not uniformly beneficial. However, these endogenous dysbiotic processes should not be regarded as established adverse effects of therapeutic H_2_ administration. Whether exogenous therapeutic H_2_ induces clinically relevant adverse alterations in the GM remains largely unknown and warrants systematic evaluation. Despite these potential concerns, the available clinical evidence generally indicates that H_2_ is well tolerated under the specific administration conditions evaluated to date. Nevertheless, this provisional safety profile should not be interpreted as evidence of unrestricted safety, because most studies involved small sample sizes, selected populations, heterogeneous delivery protocols, and relatively short follow-up periods. Route-specific maximum tolerated doses, long-term safety thresholds, and microbiota-related safety endpoints have not yet been established.

## 6 Conclusion, limitations, and future perspectives

By integrating evidence from basic, preclinical, and clinical studies, this review summarizes recent advances in the role of H_2_ in disease intervention through modulation of GM homeostasis. The GM is not only an important source of endogenous H_2_, but also a central regulator of host physiological homeostasis. Current evidence indicates that exogenous H_2_ supplementation has been associated with changes in microbial composition, intestinal barrier integrity, inflammation, and microbiota-derived metabolites. These effects may contribute to the benefits reported in metabolic, digestive, and neurodegenerative disease models. However, these parallel changes do not establish a direct causal microbiota-mediated pathway. Microbiota alterations may contribute to therapeutic effects, but they may also occur secondarily as a consequence of reduced inflammation, improved metabolism, altered intestinal physiology, or overall disease remission. Reverse causality and bidirectional interactions between host recovery and microbial ecology therefore remain plausible.

Despite increasing recognition of its therapeutic potential, the available evidence has several important limitations. As a narrative review, the present synthesis may be subject to literature-selection bias and cannot ensure exhaustive retrieval of all relevant studies. Most mechanistic studies rely on animal models with short intervention periods, whereas human studies generally have small sample sizes and limited follow-up. Heterogeneity in administration routes, H_2_ concentrations, treatment durations, diets, baseline microbial communities, and microbiome analytical methods limit cross-study comparison and external validation. Positive findings also predominate, whereas neutral and conflicting results are less frequently reported, raising the possibility of publication bias and selective outcome reporting. Accordingly, the predominance of positive published findings should not be interpreted as evidence of a consistently beneficial effect of H_2_ on the GM or clinical outcomes. Notably, a small, randomized placebo-controlled trial in ulcerative colitis found changes in microbial diversity without a clear clinical benefit, illustrating that microbiome modulation does not necessarily translate into disease improvement (Maruyama et al., 2025). The available evidence therefore supports biological plausibility and preliminary therapeutic potential rather than established clinical efficacy.

Future studies should move beyond descriptive associations toward causal and mechanistic validation. Germ-free and gnotobiotic models, microbiota depletion, FMT, single-strain reconstitution, isotope tracing, and mediation analysis may help distinguish direct H_2_ effects from microbiota-, redox-, and immune-dependent mechanisms. Intervention protocols should standardize the administration route, delivered H_2_ concentration, dose, duration, exposure measurement, diet, microbiome sampling, and safety assessment. Serial multi-omics analyses should be integrated with intestinal permeability, inflammatory, metabolic, and clinical endpoints. Large, multicenter, stratified randomized trials with preregistered protocols, adequate follow-up, transparent reporting of neutral findings, and independent validation are required to establish efficacy and identify microbial or host biomarkers of treatment response. Improved delivery systems may further enhance exposure control and reproducibility. Overall, with continued advances in mechanistic research and clinical validation, H_2_-based modulation of the GM may provide a novel avenue for the precise prevention and treatment of chronic diseases.
